# Prognostic Values of TIGAR Expression and ^18^F-FDG PET/CT in Clear Cell Renal Cell Carcinoma

**DOI:** 10.7150/jca.33442

**Published:** 2020-01-01

**Authors:** Xiaoyan Wang, Rui Li, Ruohua Chen, Gang Huang, Xiang Zhou, Jianjun Liu

**Affiliations:** 1 Department of Nuclear Medicine, Renji Hospital, School of Medicine, Shanghai Jiao Tong University, Shanghai, China; 2 Department of Ultrasound, Renji Hospital, School of Medicine, Shanghai Jiao Tong University, Shanghai, China; 3 Shanghai Key Laboratory for Molecular Imaging, Shanghai University of Medicine and Health Sciences, Shanghai, China

**Keywords:** Renal cell carcinoma, PET/CT, TIGAR, SUVmax

## Abstract

**Aim:** Evaluation of ^18^F-FDG accumulation using PET/CT is an potential imaging biomarker to reflect tumor metabolic burdens and to help predict prognosis in renal cell carcinoma (RCC). p53-induced glycolysis and apoptosis regulator (TIGAR) is a protein regulates glycolytic activity and glucose metabolism. The deregulated TIGAR expression has been associated with tumorigenesis and poor disease prognosis in several cancers. The purpose of this study is to evaluate the impact of the TIGAR expression and the maximum standardized uptake value (SUVmax) of ^18^F-FDG PET/CT on survival for patients with clear cell RCC.

**Methods:** A total of 62 patients with confirmed clear cell RCC were included in this retrospective study. The TIGAR expression of tumors were determined through immunohistochemistry staining. The SUVmax of clear cell RCC lesions were assessed using ^18^F-FDG PET/CT. The impact of TIGAR expression and SUVmax on overall survival was evaluated by the Cox proportional hazards model and the Kaplan-Meier survival analysis.

**Results:** Increased TIGAR staining was associated in clear cell RCC patients with older age, venous tumor thrombus, or increased SUVmax. A positive correlation was found between TIGAR expression and SUVmax in patients (r=0.396, *P*=0.001). Patients with positive TIGAR expression had a decreased overall survival time than those with negative TIGAR expression. The overall survival time was significantly shorter in patients with high SUVmax (>5.25) compared with those with low SUVmax (≤5.25). SUVmax and Fuhrman grade were identified as independent prognostic factors in clear cell RCC. Patients with high SUVmax (>5.25) and positive TIGAR expression were associated with a worse disease prognosis.

**Conclusion:** The expression of TIGAR is significantly correlated with SUVmax in clear cell RCC. The combined use of TIGAR expression and ^18^F-FDG PET/CT can provide additional information for tumor glucose metabolic status and disease prognosis in patients with clear cell RCC.

## Introduction

In adults, renal cell carcinoma (RCC) is the most common type of kidney cancer and accounts for about 90-95% of cancerous cases arising from the kidney 1, 2. Over the past decades, the incidence of RCC increases at a rate around 2% annually, partly due to the improved detecting ability of imaging modalities 3. Most cases are asymptomatic in clinic, often present with non-specific symptoms including weight loss and fever. Less than 15% of RCC cases present with the classic triad (hematuria, flank pain and flank mass) at the time of diagnosis and are associated with advanced disease 4. About 30% of RCC patients were reported with metastatic spread by the time of diagnosis 5. Clear cell RCC is the most common histologic type in RCC, responsible for approximately 70%-80% of RCC cases 6. Patients with clear cell RCC has been shown to have an equivalent or worse prognosis compared with other histologic types, with 5 year survival rate around 50-75% 6-8. According to previous multivariate analysis, certain factors including tumor size, Fuhrman grade, nuclear grade and components of tumor stage (T, N and M stage) were associated with disease survival in patients with clear cell RCC 7, 9-11.

^18^F-fluorodeoxyglucose positron emission tomography/computerized tomography (^18^F-FDG PET/CT) is one of the most important functional imaging modalities used for tumor diagnosis, staging, detecting metastasis and recurrence, and assessing treatment responses in various cancers 12, 13. Compared with normal tissues, malignant tumors generally have a high glycolytic activity with an adequate supply of oxygen to meet the metabolic needs for rapid tumor proliferation, which is also known as Warburg effect 14. The maximal standardized uptake value (SUVmax) is a robust metric for the assessment of FDG uptake and glucose metabolic activity of tumors *in vivo* 15. Previous studies have suggested a SUVmax≥8.8 is associated with poor prognosis in 26 patients with advanced RCC 16. Another 12 month follow-ups study also revealed an association between high disease mortality with high SUVmax (>10) in 60 patients with RCC 17. Bases on these preliminary data, it is likely that SUVmax may provide quantitative measurement of glucose metabolism of tumor lesion and predict disease prognosis in patients with RCC.

Recently, assessment of changes in molecular pathways has been shown to provide additional survival information in RCC patients. p53-induced glycolysis and apoptosis regulator (TIGAR) is a protein regulated in the p53 tumor suppressor pathways and serves an important regulatory role in the glucose metabolism of tumors 18, 19. Previous studies have suggested an association between the status of TIGAR expression and disease prognosis in patients with chronic lymphocytic leukemia, acute myeloid leukemia and non-small cell lung cancer 20-22. Moreover, p53 immunoreactivity has been recognized as a prognostic factor for RCC. Patients with p53-postive clear cell RCC was shown to have a significantly lower shorter survival rate than those with p53-negative tumors 23. It is believed that TIGAR is activated by p53 and in turn inhibits the glycolytic activity and promote pentose phosphate pathway (PPP) 24. However, to our knowledge, there is no clinical study evaluated the value of TIGAR expression in predicting disease survival in patients with RCC.

Hence, the purpose of this retrospective study is to examine the correlation between TIGAR expression and ^18^F-FDG PET/CT imaging parameters in patients with clear cell RCC. In addition, we further determine whether TIGAR expression and SUVmax could serve as predictive factors for disease survival in these patients.

## Methods

### Study population

62 patients with known RCC were recruited in this retrospective study in Shanghai Jiao Tong University affiliated Ren ji Hospital from April 2010 to June 2016. All patients received ^18^F-FDG PET/CT examination before the surgery and were confirmed to have clear cell RCC based on their histological findings. Patients were eligible for the study if (1) they did not received chemotherapy or radiotherapy before the ^18^F-FDG PET/CT examination; (2) the interval between ^18^F-FDG PET/CT scan and surgery was no more than 2 weeks; (3) complete medical records including patient demographics, clinical data and follow-up information were available; (4) surgical specimens of tumor lesions were available for immunohistochemical analysis. This study is approved by the Human Investigation Ethical Committee of Shanghai Jiao Tong University affiliated Ren ji Hospital. All patients signed the informed consent. All steps are in conformity with the Helsinki declaration.

### PET/CT imaging

All patients received a whole-body ^18^F-FDG PET/CT scan with a Biograph 64 PET/CT system (Siemens Medical Systems, German). Patients were fasted for 4-6 hours to reach a blood sugar level lower than 6.3mmol/L. Intravenous injection of 5.55MBq/kg ^18^F-FDG (radiochemical purity > 95%; provided by Shanghai Kexin Pharmaceutical Co. Ltd.) was administrated according to the body weight of patient 60min before the imaging. All patient took the supine position, and the area from the base of the skull to the middle of the femur was scanned during CT and PET imaging. CT scan was performed using voltage 120kv and current 140mA. Following parameters were applied in the PET scan: 3D model, 2min/ beds, matrix 128*128. After the image acquisition was completed after attenuation correction, ordered subset expectation maximization (2 iterations, 28 subsets) reconstruction was used to obtain PET images. The PET/CT fusion image was obtained by the Siemens post processing workstation. The image was judged by two experienced physicians of nuclear medicine. All data were imported into IntelliSpace Portal v7.0 (Philips Healthcare, The Netherlands) for automatic lesion boundaries processing. SUVmax of ^18^F-FDG uptake in lesions were automatically calculated and assessed by two experienced nuclear medicine physicians.

### Immunohistochemical staining

Immunohistochemical analyses were performed on paraffin-embedded RCC tissues. Sections (4mm slices) were obtained using microtome, and followingly processed for staining using a Nexes auto-immunostainer (Ventana Medical Systems, USA). Primary antibody against TIGAR was obtained from Abcam (1:400). Sections were assessed using a light microscope (BX51TR, Olympus, Japan) and a semi-quantitative IHC scoring system was applied to determine the intensity of TIGAR staining. For each section, 10 random areas were examined under the 200× magnification field of view using a Leica DFC320 digital camera system (Leica, Germany). A scale of 0 to 3 was applied to determine the intensity of IHC staining, as score of 0 represents the lack of brown staining, and score of 3 indicates intense dark brown immunoreactivity. Using this criterion, the properties of TIGAR expression was evaluated and scored by two experienced technicians who were blind to the condition of the patients. The intensity of TIGAR per section was then calculated and ranked as - (no staining), ± (weak brown staining), + (moderate brown staining), ++ (3 score, dark brown staining). The positive expression of TIGAR was defined as the moderate and strong staining (7). Where discrepancies occurred, the two technicians reached a consensus.

### Statistical analysis

IBM SPSS Statistics Version 20.0 (SPSS Inc, USA) and GraphPad Prism 7.0 (USA) were used for data analysis. Data was presented as mean ± standard deviation (SD). The correlations between TIGAR expression and clinicopathological characteristics were evaluated using Student's *t* test and Pearson's Chi-squared (*χ2*) test. Receiver operating characteristic curve (ROC) analysis was calculated to determine the optimal cut-off threshold for SUVmax. The correlations between TIGAR expression and SUVmax was determined using Pearson's correlation coefficients. Overall survival was calculated using by the Kaplan-Meier survival analysis, and comparisons were performed using the log-rank test. Univariate and multivariate regression was performed using the Cox proportional hazards model. All statistical tests were two-sided and a *P* value of < 0.05 was considered as statistically significant.

## Results

### Relationship between clinical characteristics and TIGAR expression

62 patients (42 men and 20 women) with confirmed clear cell RCC were included in this study. The average age of patients was 58.82±10.18 years old, ranged from 31 to 82 years old. The average survival time of patients was 41.14±27.26 months, ranged from 3 to 118 months. Among patients, positive TIGAR expression was found in tumor tissues of 38 patients (61.29%), while the rest 24 patients (38.71%) have negative TIGAR staining. In Table 1, we analyzed the relationship between the clinicopathological characteristics of patients and the TIGAR expression of tumor tissues. Patients were divided into negative and positive TIGAR expression groups based on the staining intensity of TIGAR. Increased TIGAR expression was associated with patients that older than 60 years old (*P*=0.044), with venous tumor thrombus (*P*=0.026), and with higher SUVmax (*P*=0.024). No significant correlation was found between levels of TIGAR expression and patient gender, tumor size, Fuhrman grade, lymph node metastasis or distant metastasis.

### Positive correlation between SUVmax and TIGAR expression

**Figure 1:**
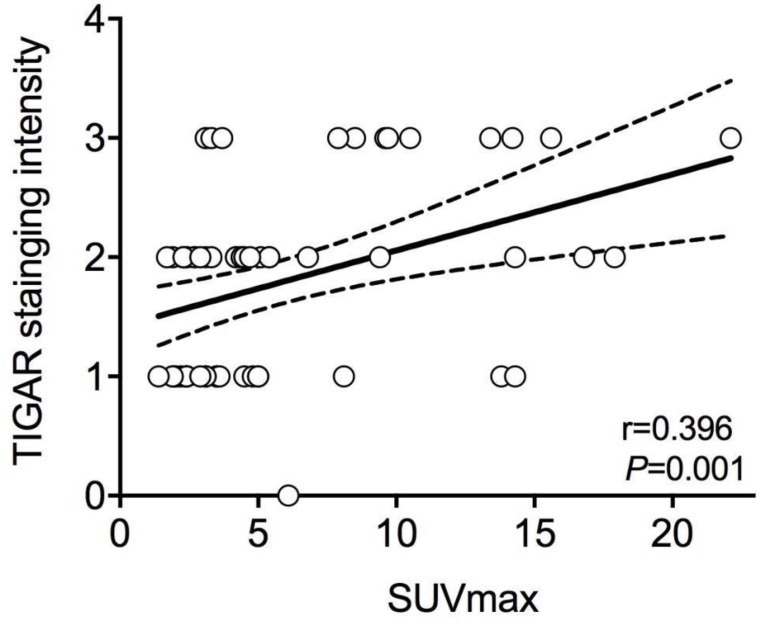
** The relationship between Pearson's correlation coefficient of TIGAR expression and maximum standard uptake value (SUVmax) in clear cell renal cell carcinoma (RCC).** A significant positive correlation is found between immunohistochemical (IHC) staining intensity of TIGAR and SUVmax (r=0.396, 95%CI=0.1621-0.5875; *P*=0.001). The solid line represents Pearson's correlation and the dotted line represents the 95% confidence bands of the best-fit line. Each circle represents one subject.

**Figure 2:**
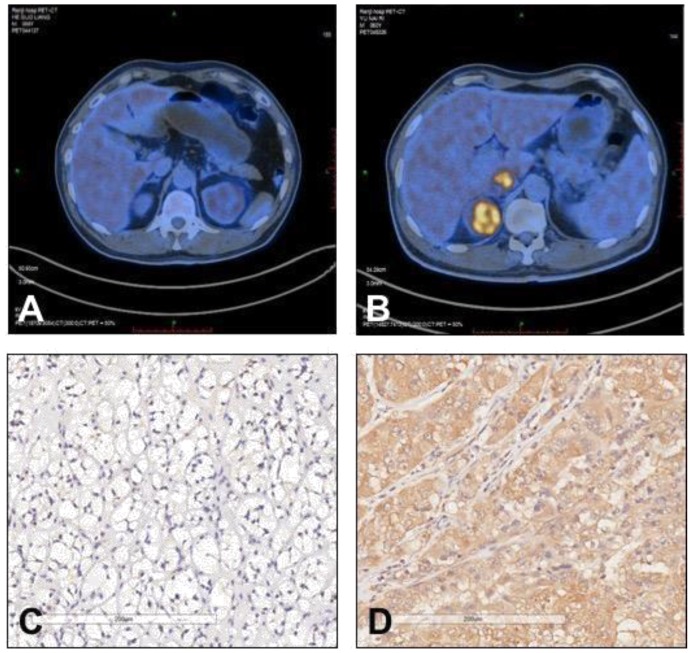
** Representative images of ^18^F-FDG uptake and immunohistochemical staining of TIGAR in patients with clear cell renal cell carcinoma (RCC). (A and C)** A 58 year old male patient had left clear cell RCC with negative TIGAR staining. ^18^F-FDG PET/CT scan did not show obvious ^18^F-FDG accumulation in the tumor lesion (SUVmax=2.4). **(B and D)** A 60 year old male patient had right clear cell RCC with positive TIGAR staining. ^18^F-FDG PET/CT scan revealed strong accumulation of ^18^F-FDG in the tumor lesion (SUVmax=9.5). Immunohistochemical images were obtained under 400× magnification.

In all patients, the average value of SUVmax was 5.83±4.78, ranged from 1.40 to 22.10. As stated above, the mean SUVmax of patients with positive TIGAR expression was significantly higher than those with negative TIGAR expression (*P*=0.024; 6.82±5.28 vs 4.26±3.40). Relationship between IHC staining intensity of TIGAR and SUVmax was then determined by Pearson's correlation coefficient (Figure 1). A positive correlation was found between positive TIGAR expression and higher SUVmax in patients with clear cell RCC (r=0.396, 95% CI=0.1621-0.5875; *P*=0.001). Representative images of TIGAR staining and ^18^F-FDG PET/CT scans of patients with positive or negative TIGAR expression are shown in Figure 2.

### Prognostic values of SUVmax and TIGAR expression in clear cell RCC

**Figure 3:**
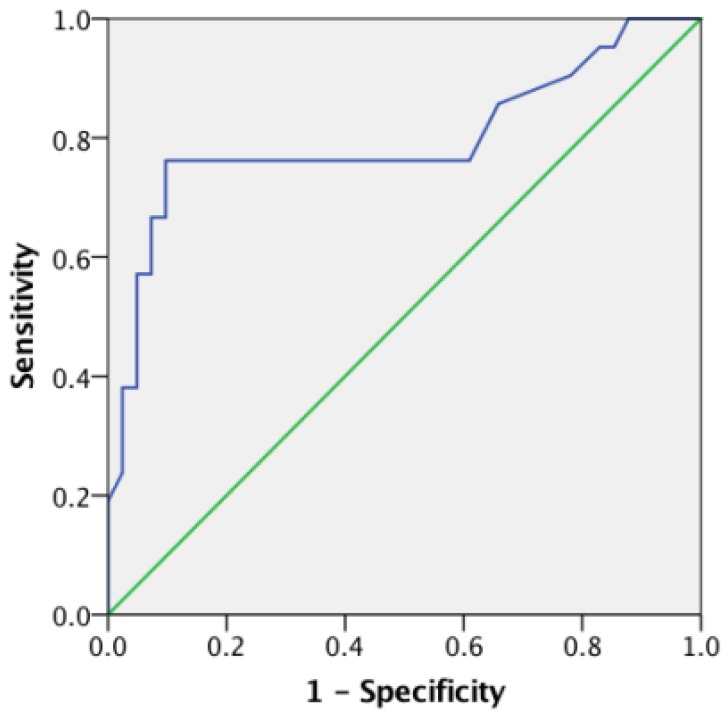
Receiver**-**operating characteristics (ROC) curve of maximum standard uptake value (SUVmax) for predicting overall survival in clear cell renal cell carcinoma (RCC). The area under the curve (AUC) is 0.796, 95% Confidence interval (CI) 0.659 to 0.933, P<0.001.

**Figure 4:**
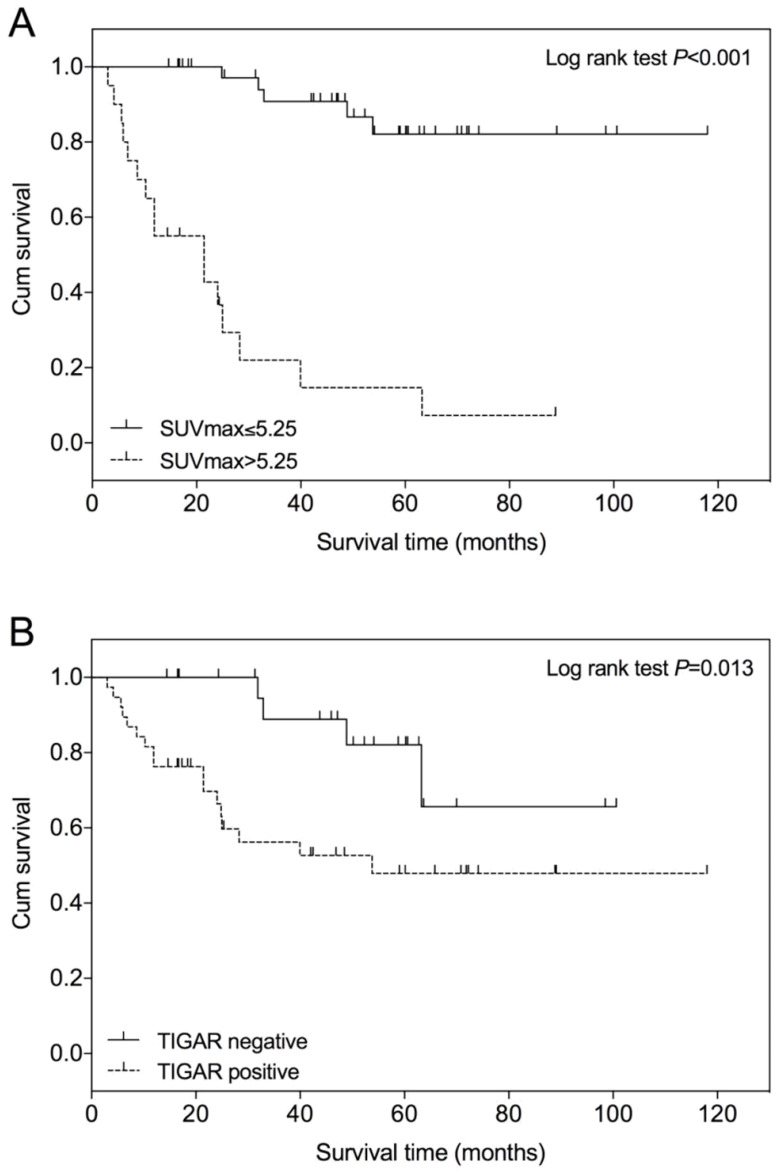
** Kaplan-Meier overall survival curve in patients with clear cell renal cell carcinoma (RCC). (A)** The survival time of patients with lower SUVmax (≤5.25) is significantly higher than those with higher SUVmax (>5.25), *P*<0.001. **(B)** The survival time of patients with negative TIGAR expression is significantly higher than those with positive TIGAR expression, *P*=0.013. The + symbol represents censored subjects.

An optimal SUVmax cutoff value of 5.25 was determined by ROC analysis for predicting survival in patients with clear cell RCC (Figure 3). The area under the curve (AUC) is 0.796 (95% CI=0.659 to 0.933; *P*<0.001). The calculated sensitivity and specificity were 0.76 and 0.90, respectively. Using this cutoff value, Kaplan-Meier survival curves were compared between patients with SUVmax≤5.25 and those with SUVmax>5.25 (Figure 4A). Survival time was significantly shorter in patients with SUVmax>5.25 (Log-rank test, *P*<0.001). Comparing the Kaplan-Meier survival curves of patients with different TIGAR expression, we found that the survival time was significantly shorter in patients with positive TIGAR expression (Figure 4B; Log-rank test, *P*=0.013).

### Univariate and multivariate analysis of the factors associated with overall survival

In the univariate Cox regression analysis, tumor size (*P*=0.008), Fuhrman grade (*P*<0.001), the presence of venous tumor thrombus (*P*<0.001), lymph node metastasis (*P*<0.001), TIGAR staining intensity (*P*=0.021) and SUVmax (*P*<0.001) were associated with overall survival in patients with clear cell RCC (Table 2). While patient age, gender, the presence of distant metastasis were not significantly associated with disease survival. All factors which were significantly associated disease survival (*P*<0.05) in the univariate analysis were then entered in the multivariate Cox regression model (Table 3). The multivariate analysis revealed that Fuhrman grade (*P*=0.032) and SUVmax (*P*<0.001) were independent factors associated with overall survival in patients with clear cell RCC.

### Prognostic value of combined SUVmax and TIGAR expression

**Figure 5:**
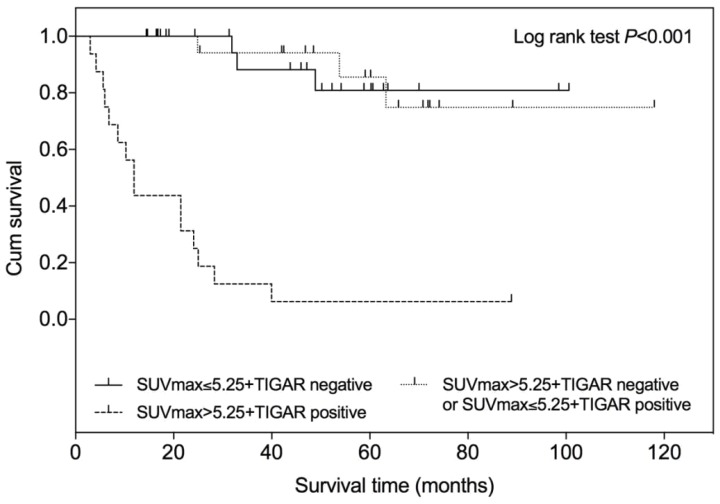
** Kaplan-Meier overall survival curve according to SUVmax and TIGAR expression in patients with clear cell renal cell carcinoma (RCC).** Higher SUVmax (>5.25) and positive TIGAR expression indicated a worse prognosis in patients with clear cell RCC when compared with other two groups (P<0.001). The + symbol represents censored subjects.

According to the SUVmax and TIGAR expression profile, all patients were divided into following three groups: patients with low SUVmax (≤5.25) and negative TIGAR expression, patients with low SUVmax (≤5.25) and positive TIAGR expression or with high SUVmax (>5.25) and negative TIGAR expression, patients with high SUVmax (>5.25) and positive TIGAR expression. The Kaplan-Meier survival analysis showed that the survival time was significantly shorter in patients with high SUVmax (>5.25) and positive TIGAR expression among three groups (Figure 5; Log-rank test *P*<0.001). No difference of survival time was observed between patients with low SUVmax and negative TIGAR expression and patients with mixed SUVmax and TIGAR profile.

## Discussion

In this study, we investigated whether the status of TIGAR expression, in addition to the SUVmax of ^18^F-FDG PET/CT, could provide additional prognostic information on disease survival in patients with clear cell RCC. The relationships between the tumor expression of TIGAR and the clinicpathological profiles in renal cancer are investigated in our study for the first time. As a result, increased TIGAR expression was found in about 60% of patients with clear cell RCC. The levels of TIGAR expression was positively correlated with tumor SUVmax of FDG uptake. Specifically, positive TIGAR expression and high SUVmax (>5.25) are associated with a poor disease prognosis in these patients, and SUVmax is recognized as an independent predictive factors for disease survival. A worse disease survival is suggested in clear cell RCC patients with positive TIGAR expression and high SUVmax.

TIGAR has been identified as a p53 target protein that serves a tumor suppressing role via regulating glycolytic activity and redox hemostasis. It shares a similar structure as phosphofructokinase 2/fructose-2,6-bisphosphatase (PFK-2/FBPase-2) and lowers glycolytic flow via degrading fructose-2,6-bisphosphate (F-2,6-P2) and inhibits phosphorfructokinase 1(PFK-1) activity 19. Considering the hallmark of reprogrammed metabolic pathways in cancer cells, it is not surprised to find dysregulated TIGAR expression in various cancer types, including lung cancer, intestinal cancer, liver cancer, and glioma 22, 25-27. Significantly elevated TIGAR expression was shown to promote tumorigenesis in primary colon cancer and metastatic sites 27. Compared to normal tissues, invasive breast cancer was associated with an higher expression of TIGAR 28. Similarly, increased TIGAR expression was found in glioblastoma 25. In our study, positive TIGAR expression was found in 61.29% (38/62) of clear cell RCC patients. The increased TIGAR protein level was associated with older age, venous tumor thrombus, and higher SUVmax in our cohort. More importantly, a shorter survival time was found in clear cell RCC patients with positive TIGAR compared with those with negative TIGAR expression (HR=3.620, 95%CI 1.214-10.795). Previous studies have suggested that TIGAR plays a role in tumor progression by regulating tumor burden and redox homeostasis 19. Knock down of TIGAR expression increases radiotherapy sensitivity in glioma cells by increasing ROS accumulation and inducing DNA damage 25. In another study using a TIGAR deficient mouse model, decreased TIGAR expression reduced colon tumor burden and size, resulting in a better survival outcome 27. Together, these results indicate that TIGAR servers as an important indicator in the tumorigenesis and prognosis of clear cell RCC.

^18^F-FDG uptake, as determined by SUVmax, is a good predictive imaging biomarker for disease survival in various cancer types. As a result, a SUVmax cutoff of 5.25 was determined in this study by ROC analysis with a sensitivity of 0.76 and specificity of 0.90. Using univariate analysis, tumor size, Fuhrman grade, venous tumor thrombus, lymph node metastasis, SUVmax and TIGAR expression are shown to be associated with disease survival in clear cell RCC. Furthermore, the multivariate analysis revealed SUVmax and Fuhrman grade as two independent predictive factors for overall survival in patients with clear cell RCC. These findings are in good consistent with previous reports 7, 9, 16. A SUVmax cutoff of 8.8 was identified in a preliminary study includes 26 patients with advanced or metastatic RCC. They found that patients with higher SUVmax were associated with a poor prognosis (HR=1.326, 95% CI 1.089-1.614), as well as identifying SUVmax as an index to predict the survival time of patients with advanced RCC 16. Another study also reported a correlation between higher SUVmax (>10) and an increased mortality rate in 60 RCC patients 17. In our study, clear cell RCC patients with a higher SUVmax (>5.25) had a significantly decreased overall survival time (HR=5.273, 95% CI 1.914-14.527).

In normal cells, the expression of TIGAR level is tightly regulated during different stages of p53 induced cell death. The TIGAR level first upregulated during the initial stage of p53 induced cell stress, and then drops when the cells are under a transition towards an apoptotic state 19. Interestingly, contrary to its function in the p53 tumor suppression pathway, our study revealed a significant correlation between increased TIGAR expression and increased SUVmax in patients with clear cell RCC. It has been suggested overexpressed TIGAR may exert a tumor promoting function uncoupled from p53 expression 27. TIGAR is known to bind with and active the rate-limiting glucose metabolic enzyme hexokinase 2 (HK2), and in turn increases the production of metabolic intermediates and promotes cell proliferation via PPP 29, 30. Given the well documented Warburg effect, most tumor cells exerts an upregulated aerobic glycolysis and glucose metabolism under adequate oxygen supply. Elevated SUVmax of ^18^F-FDG suggested an enhanced glycolytic activity and glucose metabolism in tumor cells, which is associated with the upregulation of glycolytic enzymes including HK2 31. According to the upregulated TIGAR expression along with the high SUVmax in our results, it is possible that an upregulated glycolytic and PPP activity may occur in these patients with clear cell RCC. Consistent with our findings, a recent *in vivo* isotope tracing study revealed a significantly increased enrichment of glycolytic intermediates and a lowest TCA cycle intermediate enrichment, suggesting enhanced glycolysis and suppressed mitochondrial oxidation in clear cell RCC 32. It is worthy to notice that correlation between TIGAR expression and SUVmax can vary depending on different cancer types, and may serve as a potential indicator for glucose metabolic phenotypes. Unlike clear cell RCC with an enhanced glycolysis and inhibited phosphorylation oxidation, studies revealed a preserved mitochondrial oxidation function in human lung cancers 33, 34. Consistently, our previous study also revealed an opposite relationship between TIGAR expression and SUVmax in patients with lung cancers, as negative TIGAR expression was significantly associated with higher SUVmax 22. Based on our findings and previous publications, the combined use of SUVmax and TIGAR may provide additional information regarding the changes of glucose metabolic flux in different types of tumors.

The present study has a few limitations. Firstly, the retrospective study design may subject to the selection bias. Secondly, a relatively small number of patients was included in this study, which also may contribute to selection bias. Thirdly, due to the limited sample size, it is difficult for us to have a separate patient cohort to validate the major findings. We currently are planning to conduct a perspective clinical study in collaboration with urology surgeons to collect more renal cancer cases to better address and validate the prognostic value the TIGAR expression and SUVmax in renal cancer.

In conclusion, our study is so far the first report evaluated the prognostic value of TIGAR expression and SUVmax in patients with clear cell RCC. SUVmax and Fuhrman grade are found to be independent predictors for prognosis. A positive TIGAR expression was correlated with increased SUVmax. Patients who have positive TIGAR expression and high SUVmax were associated with a worse disease overall survival. Combined use of TIGAR expression and ^18^F-FDG PET/CT may provide additional information to help evaluate glucose metabolism and disease prognosis in clear cell RCC.

## Figures and Tables

**Table 1:** Baseline characteristics of patients and univariate analysis for overall survival (N=62)

Variables	Subgroups	TIGAR Expression		P value
		Low (-~±)	High (+~++)	
**Age**	≤60yrs	17	17	0.044
	>60yr	7	21	
**Gender**	Male	17	25	0.679
	Female	7	13	
**Tumor size**	≤4cm	12	14	0.306
	>4cm	12	24	
**Fuhrman Grade**	Low	20	25	0.131
	High	4	13	
**Venous Tumor**	No	23	28	0.026
**Thrombus**				
	Yes	1	10	
**Lymph Node**	No	20	29	0.509
**Metastasis**				
	Yes	4	9	
**Distant**	No	20	29	0.509
**Metastasis**				
	Yes	4	9	
**SUVmax**		4.26±3.40	6.82±5.28	0.024

**Table 2:** Univariate COX regression analysis of overall survival in patients with clear cell renal cell carcinoma (N=62)

Variables		Univariate analysis	
	HR	95% CI of HR	P value
Age	1.486	0.627-3.517	0.368
Gender	0.846	0.350-2.045	0.711
Tumor Size	4.348	1.458-12.964	**0.008**
Fuhrman Grade	6.603	2.707-16.107	**0.000**
Venous Tumor Thrombus	5.835	2.235-15.233	**0.000**
Lymph Node Metastasis	4.887	2.035-11.737	**0.000**
Distant Metastasis	2.378	0.956-5.919	0.063
TIGAR Intensity	3.620	1.214-10.795	**0.021**
SUVmax	5.273	1.914-14.527	**0.001**

HR: hazard ratio; CI: confidence interval HR: hazard ratio; CI: confidence interval.

**Table 3:** Multivariate COX regression analysis of overall survival in patients with clear cell renal cell carcinoma (N=62)

Variables		Multivariate analysis	
	HR	95% CI of HR	P value
Tumor Size	0.397	0.043-3.638	0.414
Fuhrman Grade	0.222	0.056-0.881	**0.032**
Venous Tumor Thrombus	1.410	0.338-5.887	0.637
Lymph Node Metastasis	1.641	0.402-6.698	0.490
TIGAR Intensity	2.129	0.654-6.933	0.210
SUVmax	78.93	15.921-391.321	**0.000**

## Abbreviations

^18^F-FDG PET/CT: ^18^F-fluorodeoxyglucose positron emission tomography/computerized tomography
F-2,6-P2: fructose-2,6-bisphosphate
HK2: hexokinase 2
IHC: Immunohistochemistry
FBPase-2: fructose-2,6-bisphosphatase
PFK-1: phosphofructokinase 1
PFK-2: phosphofructokinase 2
PPP: pentose phosphate pathway
ROC: receiver operating characteristic curve
RCC: renal cell carcinoma
SUVmax: maximal standardized uptake value
TIGAR: p53-induced glycolysis and apoptosis regulator
